# Using Spectral-Domain Optical Coherence Tomography to Follow Outer Retinal Structure Changes in a Patient with Recurrent Punctate Inner Choroidopathy

**DOI:** 10.1155/2011/753741

**Published:** 2011-07-10

**Authors:** Kimberly E. Stepien, Joseph Carroll

**Affiliations:** ^1^Department of Ophthalmolog, Eye Institute, Medical College of Wisconsin, 925 N 87th Street, Milwaukee, WI 53226, USA; ^2^Departments of Cell Biology, Neurobiology, Anatomy, and Biophysics, Medical College of Wisconsin, Milwaukee, WI 53226, USA

## Abstract

Punctate inner choroidopathy (PIC) is a rare idiopathic inflammatory disorder of the retina and choroid usually affecting healthy, young, myopic females and presenting with photopsia, paracentral scotomata, and blurred vision. It is characterized by yellow-white chorioretinal lesions concentrated in the posterior pole, no vitritis, relapsing inflammatory activity of the retina and choroid, and frequent development of choroidal neovascular membranes. Here we describe a case in which spectral-domain optical coherence tomography (SD-OCT) imaging was used to monitor outer retinal structure changes associated with recurrent PIC over time. SD-OCT, which is both quantative and objective, provides an efficient, non-invasive way to follow recurrent inflammatory chorioretinal lesion activity, choroidal neovascular membrane development, and treatment response in patients with recurrent PIC.

## 1. Report of a Case:

A 21-year-old white myopic female with a history of symptomatic punctate inner choroidopathy (PIC) presented with new photopsias and scotoma in her left eye for several days. She had been symptomatic in her right eye for one year with documented new chorioretinal lesion formation and development of a choroidal neovascular membrane (CNVM) successfully treated with photodynamic therapy (PDT), intravitreal triamcinolone, and intravitreal bevacizumab (Avastin, Genentech, South San Francisco, Calif). Past ocular history was significant for herpetic keratitis for which she was taking acyclovir 400 mg daily.

**Figure 1:**
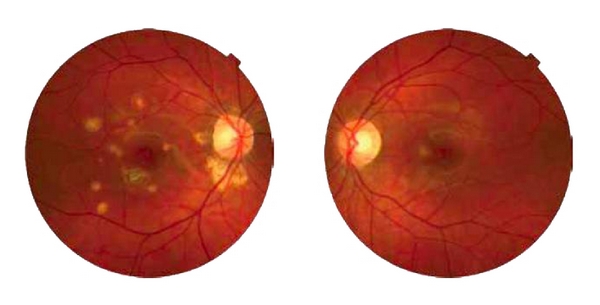
Right and left fundus photos of patient with recurrent punctate inner choroidopathy. Right fundus shows multiple yellowish-white chorioretinal lesions, some appear atrophic, localized to the posterior pole. Just inferior to the fovea is a pigmented scar from a successfully treated choroidal neovascular membrane. The left fundus shows a new focal yellowish chorioretinal lesion with surrounding fluid not documented on previous fundus photographs.

On exam, visual acuity was 20/20 OU with myopic correction. Anterior chamber and vitreous were without inflammation. Funduscopy showed multiple yellowish-white chorioretinal lesions and a pigmented scar from the CNVM localized within the posterior pole in the right eye, and a focal chorioretinal lesion with fluid just nasal to the fovea in her left eye (Figure 1). When compared to previous fundus photos, the lesion in the left eye was new. No peripheral lesions were present. Fluorescein Angiogram (FA) showed focal leakage, left eye, consistent with CNVM. Indocyanine green (ICG) showed hypofluorescent spots corresponding to the chorioretinal lesions consistent with PIC [1] in both eyes, and a focal area of hyperfluorescence at the edge of the hypofluorescent spot in the left eye, suggesting a CNVM.

**Figure 2:**
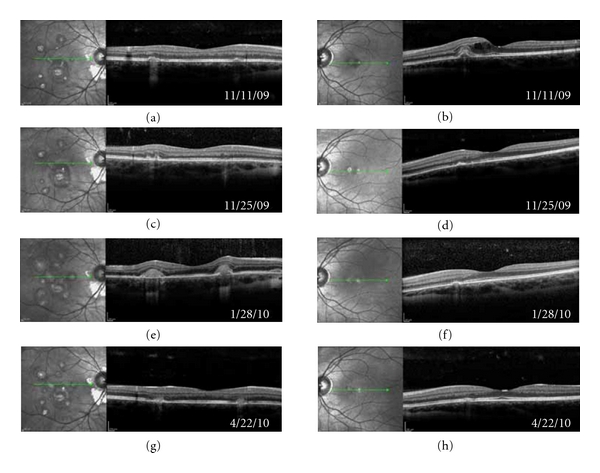
Spectralis spectral-domain optical coherence tomography (SD-OCT) images taken at the same location of both eyes over a 5.5-month period in a patient with recurrent punctate inner choroidopathy. (a) and (b) Initial visit. (b) Shows outer retinal irregularity and inner retinal fluid (IRF) from choroidal neovascular membrane (CNVM). (c) and (d) Followup two weeks later. After intravitreal bevacizumab treatment, left eye, shows resolution of IRF (d). (e) and (f) Two months later shortly after stopping chronic antiviral therapy. The patient experienced new photopsias, right eye, and SD-OCT revealed homogenous outer retinal thickening over chorioretinal lesions consistent with recurrent inflammatory activity (e). (g) and (h) Three months later after treatment. Symptoms subsided, and outer retinal thickening has resolved (g). Left eye shows no reoccurrence of CNVM (h).

Spectralis spectral-domain optical coherence tomography (SD-OCT) (Spectralis HRA; Heldelberg Engineering, Heidelberg, Germany) of the left eye at the initial visit showed retinal pigment epithelium (RPE) elevation with surrounding intraretinal fluid (IRF) consistent with CNVM (Figure 2(b)). Two weeks after treatment with intravitreal bevacizumab, SD-OCT showed resolution of IRF (Figure 2(d)).

Over the next 2 months, the patient continued to have symptomatic photopsias in both eyes. Clinical exam continued to show no anterior chamber inflammation or vitritis. She was placed on an extended oral prednisone taper. While still on 30 mg of oral prednisone, she experienced an acute increase in photopsias in her right eye. Four days earlier, she had run out of oral acyclovir. SD-OCT was suggestive of recurrent inflammatory lesion activity with new homogeneous outer retinal thickening overlying chorioretinal lesions but with no IRF (Figure 2(e)). The patient was treated with a higher dose of oral prednisone, oral valacyclovir (Valtrex, GlaxoSmithKline, Research Triangle Park, NC, USA), and two intravitreal bevacizumab injections over the next 2 months. Her symptoms resolved, and SD-OCT showed improvement in thickening over the chorioretinal lesions. Vision is still 20/20 OU.

## 2. Discussion

First described by Watzke et al. in 1984, PIC is a rare idiopathic inflammatory disorder of the retina and choroid usually affecting healthy, young, myopic females and presenting with photopsia, paracentral scotomata, and blurred vision [2]. Initial symptoms are usually unilateral although exam shows bilateral disease [2, 4]. Clinically, yellow-white chorioretinal lesions ranging in size from 100 to 300 microns are concentrated in the posterior pole and there is no vitreous inflammation [3]. 

Vision loss with PIC is usually secondary to development of CNVMs which can occur in 40–76% of patients [2–5]. Recently, intravitreal anti vascular endothelial growth factor (anti-VEGF) agents has been shown to be very effective in the treatment of CNVM associated with PIC [6–8]. SD-OCT imaging in our patient documented a great treatment response after intravitreal bevacizumab injection with resolution of IRF just 2 weeks after treatment. 

PIC is characterized by relapsing inflammatory activity of the retina and choroid [9]. Unlike with CNVM where IRF was visualized, SD-OCT showed a homogenous thickening over the chorioretinal lesions with recurrent inflammatory activity. In this patient, her recurrent PIC may be associated with a viral etiology, as symptoms increased and SD-OCT documented outer retinal changes suggesting recurrent inflammatory activity shortly after cessation of chronic antiviral therapy. After restarting both immunosuppressive and antiviral therapy, both symptoms and SD-OCT findings improved. 

SD-OCT gives excellent detail of outer retinal structures in patients with PIC. In this patient with recurrent PIC, SD-OCT imaging provided a quantitative and objective way to monitor outer retinal structure changes associated with CNVM development, treatment response, and recurrent inflammatory chorioretinal lesion activity. Given it is noninvasive, SD-OCT may prove to be a very effective way to monitor and better understand pathology in patients with PIC who develop CNVM and/or recurrent inflammatory activity.
